# A Notice of Cholera as It Appeared in Louisville in May, 1849

**Published:** 1849-06

**Authors:** Theodore S. Bell


Art. V.—*A Notice of Cholera as it appeared in Louisville in May* 1849. By Theodore S. Bell, M.D.

We hasten to lay before our readers a brief history of epidemic cholera as it visited our city during the month just closed, and in doing so shall avail ourselves of the Report of the Committee of the Board of Health, as follows:

At a meeting of the board of health of Louisville, on the 25th inst., the undersigned were appointed to draw up a sketch of the recent epidemic cholera in this city. In the discharge of that duty, we have endeavored to investigate the subject very fully, and the following is the result of our investigations:

On the 2d of May, it was announced that on the preceding day several cases of cholera had occurred between Main and water streets, as the northern and southern boundaries, and between Fifth and Sixth as the eastern and western limits. The first case was immediately below the summit of the second bank of the river, and in the vicinity of yards that had received the washings of the ascending ground above them, and which were otherwise not by any means notable for cleanliness. It has been noted, too, as a somewhat remarkable occurrence, that the first cases of 1832 and 1833 were in the same square with those of 1849.

To resume, however, the sketch of the progress of cholera, on the 2d of May three deaths from the disease were announced, and some four or five cases were reported as recovering. On the 3d of May, three deaths were reported as having occurred on Fifth street, between Main and Water, and one death in an alley in the vicinity. On the 4th of May, several new cases were reported and two deaths. It was remarkable, at the time, that the most of these miserable victims had died without having sought or received any kind of medical attention. On Saturday, the 5th, the reporter for the Louisville Courier published that his dilligent inquiries, up to a late hour of Friday night, had not enabled him to hear of a death in the preceding twenty-four hours. From Friday, the 4th of May, up to Monday, the 7th, four deaths were reported from cholera. On the 8th, five deaths were announced. On the 9th, three new cases were reported, but no deaths. On the 10th, several new cases and three deaths. On the 12th, the statement was published, on the authority of a number of physicians, that there was not a case on the preceding day, but on the night of the 13th, it was stated, there were three deaths, two of whom were negroes. On Monday, the 14th, the statement was published that four deaths had occurred since the preceding Friday night, and it was also announced that the disease was still circumscribed in a very limited range. Among the four deaths thus announced, is included that of Mr. D. Marble, who had arrived here on the preceding Friday evening from St. Louis, with the premonitory symptoms of the disease while on the boat. During all this time, the general health of the city was reported, though there was a great tendency to diarrhea, on the authority of the physicians, to be good. On the 15th, six new cases were announced, and all recovered, except a negro on the bank of the great ditch, in the southern part of the city. From this up to the 21st, diligent inquiry, made daily, resulted in stating that not a death from cholera had been heard of; and on the morning of the 21st, the Morning Courier stated that "there had not been a single death from cholera in Louisville during the week ending at 10 o'clock the preceding night."

On the 22d, a German woman near the river died, and on the 23d and 24th, it was stated that not a case had been heard of in either of those days. On the 24th, the Courier announced that in consequence of the appointment of a board of health by the mayor and council, it should cease making reports on cholera.

On the afternoon of the 25th, the board of health met and reported that they knew of no cholera in the city, and that the general health of the city was remarkably good.

In the published proceedings of the board of health, a request was made of the physicians of the city that they should report all cases of cholera occurring in their practice, to the board of health, and the ordinance requires "that the sexton of the city graveyard shall report daily to the board of health, the interments in said yard, and that the resident physician of the hospital, and the physician to the poor and pest houses shall report daily, to the board, the number of cases of cholera and of deaths therefrom in each institution, and all the particulars of the same." From no one of these sources of information have the board of health heard anything on the subject of cholera, nor have they any reason to suppose that there was anything to report to the board.

They are, therefore, gratified in being able to say that the health of Louisville continues good, and that they know of no death from cholera since their preceding report.

Here, then, we have the remarkable fact in testimony of the healthiness of Louisville, that in the course of twenty-five days, the period of the existence of cholera in this city, in a population of about fifty thousand persons, there were but thirty-three deaths. It may be supposed that all the deaths were not reported, nor are we able to say they were, but the gentlemen whose special duty it was to attend to this business were zealous, diligent, and faithful in the discharge of their duty, and they spared no pains to be accurate and full in their statements. We know that some of the cases enumerated above, were not cases of cholera, and if we make these an offset to possible omissions of some deaths, we shall probably be near the truth.

It is gratifying to know that the intelligent use of those faculties which Providence has given to man, may enable him to guard against the ravages of the destroying pestilence, and that "the evil which springs from the bosom of Nature, only needs for its removal an observance of the rules which Nature herself reveals." We have ample reason, while bowing with grateful hearts to that Providence that has guided us through the pestilence, to encourage us to a steady adherence to those sanitary measures which have been most useful to us. If we maintain the exemption, we have thus far experienced, from cholera, we must maintain that state of things which acts as preventive means.

THEODORE S. BELL,
L. POWELL,
LEWIS ROGERS.

It may be deemed necessary that we should say something of the treatment of cholera in this city. The physicians of Louisville, in addition to numbers of sporadic cases, have had three visitations of endemic cholera, and they have been very successful in the management of the disease. Instead of flying to specifics, they have endeavored to meet the malady upon those principles which science, common sense, and experience have inculcated. The scientific physician, when about to prescribe for a patient must take in an immense field of vision that is unknown and disregarded by the mere routinist. He must, in making up his opinion, take into consideration THE AGE of the patient, and the modifying influence of social condition—THE SEX, and the habits of life — TEMPERAMENT, CONSTITUTION, PECULIARITIES, etc. The present condition of the patient—general appearance, strength, etc. The entire character of the nervous system — general and special sensibility—pain, its characters and intensity—state of all the senses—the condition of the intellect—sleep. THE DIGESTIVE APPARATUS—appetite—degree, perversions, thirst. The tongue, size and shape, color, coat, moisture—nausea, matters vomited—size of abdomen, pain, sensibility on pressure. STOOLS, their frequency and quality. Dimensions, etc., of liver, spleen, kidneys, and bladder. The condition of the RESPIRATORY ORGANS—circulatory organs—state of the SKIN, and of the EXCRETIONS.* These are merely the heads of the topics which every physician must pass in review in making up a scientific prescription, and each of these heads of a subject has various tributary streams, which must be fully explored in forming a judgment as to the condition of the patient. This is but the beginning of the work: the resources of

* See Stillé's Elements of Pathology.

the Materia Medica must then be considered, and the best means selected for fulfilling the indications arising from the pathological views already referred to. And chemical knowledge must be at hand, to provide against the admission of incompatible substances into the prescription.

These mental processes are unseen by the spectator, and are unknown to all but the physician. The immense interests at stake show the importance of relying upon men whose studies, acquirements, and experience, qualify them to prosecute these investigations. It is evident, from these remarks, that while the physician cannot be too well qualified, the patient cannot be too careful as to whose hands he commits his life. Hence, too, the folly of relying upon any specific remedy for any disease—the varying phases of all maladies, must be met by corresponding variations in the remedies used. But it is astonishing with what avidity people swallow all kinds of stuff, when they are frightened by the contiguity of cholera. Humanity shrinks into amazingly small proportions while under the dominion of fear, and men, in their oscillations from the paths of common sense, are much more certain to do wrong than right. They indulge the vain hope that they may guard against the approaches of a powerful malady, by doing all they can to invite its attack. They dismiss the sentinels of reason from their posts, and wander over a trackless waste of darkness without compass or guide. They swallow brandy, opium, lead, and other noxious agents without sense, reason, discretion, or discrimination.

Whenever a terrific and sweeping pestilence commences its ravages, it is surprising to see what flocks of cormorants wing their way to the scene of devastation, to prey upon the credulity, the fears, and the follies of the public. With the most imperturbable mendacity, the coolest impudence imaginable, and the most utter indifference to all consequences, they announce that they have an unfailing remedy for the disease; that it is suitable for every age, condition, and sex, and for any stage of the distemper, and that its miraculous powers have been hidden from all the human race, but the particular individual who manufactures it. They announce that it succeeds where physicians fail, and that nothing but the purest philanthrophy induces them to keep it secret, and to offer it for sale to a suffering world. These things are an utter abomination, and demand the interposition of law, not to protect medical men, but the public, from these designing sharpers. They do infinite harm, and can do no kind of good.

In the midst of a great conflagration, or near the scene of a wreck, we see philanthropists of the same stripe we have described as hovering over the ranges of a destroying pestilence. These characters are busily engaged in saving all they can lay hold of from the devouring elements, and are filled with an amazing love for the human race, whose entire boundaries are contained in—self.

In the report of the board of health, the statement is made that the miserable victims of cholera in the neighborhood of the river died without seeking or receiving any medical attention. Each Samaritan who rushed to the scene of misery went armed with his particular nostrum, with which he advised the patient to combat a malady, that often baffles all the resources of medical art, and the stomach was filled with the frightful products of a heartless empiricism, that cared for nothing beyond meddling with what it knew nothing about.

In the generality of cases treated by the physicians of Louisville, the treatment has been attended with the most gratifying success. The rate of mortality has been unusually small, and it has been so on account of the judicious, energetic, and discriminating character of the remedies employed. These have varied according to the circumstances we have named, as operating upon every scientific physician in making up his judgment upon each individual case. No routine system of treatment has been resorted to among the physicians of this city.

The plan usually adopted in the very early stages, is to give calomel, in doses varying from ten grains to ℈j, combined with small portions of powdered opium, black drop, or laudanum. Sometimes the saccharum saturni is added. If there is great irritability of the stomach at this stage it is combatted best with small portions of calomel and black drop, given at short intervals. In some cases where calomel, in doses of ten grains every two hours, was given with one grain doses of opium, and where the failure to relieve was decided, we have seen the best results flow from giving calomel in three grain doses, combined with three drops of the acetated tincture of opium, every fifteen or twenty minutes. Some cases have been saved in this way that were rapidly running into collapse, under the use of larger doses given at greater intervals. We think, however, that the opium should be used with all possible moderation, and that its employment should be dispensed with as soon as it can be with safety to the patient. We prefer doing without it in all cases where it can be safely omitted. The free use of it in cholera is not attended with a very gratifying control over the disease, nor with any very flattering results in the stage of convalescence. But we consider calomel the sheet-anchor of the treatment, and feel sure, reports to the contrary notwithstanding, that no other treatment of cholera has ever been so successful. In a conflict between the testimony of others, and the evidence of our own senses, we unhesitatingly prefer believing the latter. We have heard much vaunting of the triumphant use of other means than mercurials, and we have seen many efforts at doing without calomel in the treatment of cholera, but we have yet to see the first evidence of a result that would tempt us to lay aside its use. That this great remedy is often used improperly, indiscreetly, and disastrously, is certainly true, but these facts are not to be considered as having any bearing upon the proper use of the medicine. It is contrary to the rules of philosophy to argue against the use of anything on account of the abuse of it. Nor are we compelled to believe all we hear or read. An ample experience in the cholera of 1832, 1833, and 1849 in Louisville, furnishes us with the most conclusive evidence of the safety and value of the remedial powers of calomel, nor do we know of anything that shakes the proof. We are not wedded to that remedy, and when we see anything that is to be compared with it, for a controlling influence over cholera, we shall cheerfully adopt it. The proximate cause of cholera—that which when present causes the disease, which when changed, changes it, and which when removed, removes it, is an inactivity of the liver, kidneys, and skin, and great irritability of the stomach and bowels. In the whole range of the Materia Medica we know of nothing that exerts the power, in this state of things, that calomel does, when combined with small portions of opium or black drop. The enlightened physician will always remember that he is using powerful means to subdue disease, not the patient, and he will cease the use of such means at the earliest moment he can, with safety to the patient. Calomel is essential in the treatment of cholera, but it is not necessary to use it in such enormous quantities as are sometimes given. In not a limited experience, we declare, that we have never seen a case of undoubted cholera recover without the administration of calomel, nor have we ever seen any disastrous consequences as the result of its use. We do not undertake to say what others have seen; we speak only of our own experience.

In the initial stages of cholera, of which we have thus far spoken, the recumbent posture, steadily maintained, is a *sine qua non;* the feet should be bathed in mustard water, and the abdomen should be covered with a mustard plaster. In some obstinate cases of diarrhea and suppresion of urine, we have seen uniform good effects from the application of mustard to the spine, throughout its entire length.

But it is not often the good fortune of the physician to see a case at this early period of the development of the disease. He is not often called in until the vomiting, diarrhea, and spasms have become very urgent. At this stage the powers of life are rapidly waning; the fluids are in swift retreat from the surface to the interior of the body; the skin is cold and clammy; the pulse is sinking; the respiration is labored; and everything denotes that collapse is approaching. In this state of things whatever is done must be done quickly and with energy.

The course found most successful here, when collapse is approaching, is first: the extensive application of sinapisms of mustard—they should be applied dry to the feet and hands, arms and legs. The mustard poultice to the spine and abdomen is invaluable, and lumps of unslacked lime, wrapped in *wet* cloths, laid around the patient underneath the bed-clothes, have a speedier and finer effect than any other method of applying heat we have ever known. In the most of such cases there is no time to lose in waiting for hot bricks, nor are bottles of hot water very eligible. The lime, wrapped in wet cloths, commences its activity immediately, and we have seen it restore the capillary circulation in cases where its return seemed utterly hopeless.

These are the first and most important methods of treatment in these desperate circumstances. The next thing to be done is to administer calomel in from ten to twenty grain doses, with black drop in doses of ten or fifteen drops, and thirty drops of camphor dissolved in chloroform. The latter is made by dissolving ʒiij of powdered camphor in ʒi of chloroform. It is rarely necessary to repeat the camphor and black drop, and we have rarely ever seen any remedy that exercised a more certain or more potent influence over congestion than this camphor and chloroform. We have repeatedly seen this solution rouse the system from the profoundest torpor, and in which we have uniformly met with failure in the use of all other means.

After the capillary circulation is once more in healthful action, the liver must be relieved by those judicious measures which are in the hands of all intelligent practitioners. It should never be forgotten that the resources of medical science are ample for the control of cholera, and if they are properly applied, they cannot fail to triumph over this deadly distemper.

We intended to give a detail of several interesting cases that have occurred in this city, but we find that we have not sufficient space for them in this department of the Journal, for this month. We shall endeavor to present them to our readers in the July number, in order to illustrate the value of the means of cure we have indicated.

We will only add, that since the report of the board of health, on the 25th, no death from cholera in the city has come to our knowledge.

May 31, 1849.

